# Multidimensional Approach for Investigating the Effects of an Antibiotic–Probiotic Combination on the Equine Hindgut Ecosystem and Microbial Fibrolysis

**DOI:** 10.3389/fmicb.2021.646294

**Published:** 2021-03-25

**Authors:** Axelle Collinet, Pauline Grimm, Samy Julliand, Véronique Julliand

**Affiliations:** ^1^Lab To Field, Dijon, France; ^2^Univ. Bourgogne Franche–Comté, AgroSup Dijon, PAM UMR A 02.102, Dijon, France

**Keywords:** large intestine, microbiota (microorganism), horse (Equus caballus), dysbiosis, immune response, bacterial functional groups, 16S rRNA gene sequencing analysis

## Abstract

The equine hindgut ecosystem is specialized in dietary fibers’ fermentation to provide horses’ energy and contribute to its health. Nevertheless, antibiotics are known to disrupt the hindgut microbiota, affecting the fibrolytic activity of bacteria and the intestinal immune balance, leading to diseases. This *in vivo* study used a general and comprehensive approach for characterizing the hindgut ecosystem of 9 healthy horses over 28 days in response to a 5-day challenge with oral trimethoprim-sulfadiazine (TMS), with a special emphasis on microbial fibrolytic activity and the host immune response. Horses were supplemented with two doses of *Lactobacillus acidophilus*, *Ligilactobacillus salivarius* (formerly *L. salivarius*), and *Bifidobacterium lactis* blend or a placebo in a 3 × 3 Latin square design. Changes in fecal microbiota were investigated using 16S rRNA sequencing. *Clostridioides difficile* was quantified in feces using quantitative polymerase chain reaction. Anaerobic microbiological culture was used to enumerate functional bacterial groups (cellulolytic, amylolytic, and lactic acid-utilizing). The environmental dimensions were assessed by measuring the concentrations of volatile fatty acids (VFAs) and lactic acid using biochemical methods, and changes in pH and dry matter weight. Systemic and local inflammation was evaluated by determination of cytokine and immunoglobulin (Ig)A concentrations in the serum and secretory IgA (SIgA) concentrations in the feces using immuno-enzymatic methods. Oral TMS treatment strongly altered the whole hindgut ecosystem by 2 days after the first administration. Bacterial diversity decreased in proportion to the relative abundance of fibrolytic genera, which coincided with the decrease in the concentration of cellulolytic bacteria. At the same time, the composition of microbiota members was reorganized in terms of relative abundances, probably to support the alteration in fibrolysis. *C. difficile* DNA was not found in these horses, but the relative abundances of several potential pathobiont genera increased. 2 days after the first TMS administration, fecal concentrations of VFAs and SIgA increased in parallel with fecal water content, suggesting an alteration of the integrity of the hindgut mucosa. Recovery in bacterial composition, functions, and immune biomarkers took 2–9 days after the end of TMS administration. Supplementation with this bacterial blend did not limit bacterial alteration but might have interesting mucosal immunomodulatory effects.

## Introduction

The equine hindgut ecosystem is made up of all the living and non-living components of the large intestine acting in complex and constant interactions. The biotic component of this ecosystem, collectively called the microbiota, is essential for ensuring fiber degradation in the equine hindgut. This is mainly because of the fibrolytic activity of several keystone microorganisms that have the capability to break down indigestible parietal carbohydrates and produce volatile fatty acids (VFAs). VFAs are a major source of energy and provide half or more of the horse’s digestible energy requirements (Julliand and Grimm, 2016). Among several environmental factors in equine management that are known to modulate the diversity and composition of the hindgut microbiota and also influence fibrolytic activity, antibiotic administration has been identified as important. In horses under oral trimethoprim sulfadiazine (TMS) or intramuscular sodium ceftiofur treatments, a drastic decrease in fecal cellulolytic bacteria has been observed during the week of treatment and in the following week of withdrawal. This decrease was associated with a concomitant decrease in lactobacilli and simultaneous increases in two intestinal pathogens, *Salmonella* and *Clostridioides difficile*, formerly *Clostridium difficile* (Lawson et al., 2016), in the feces of healthy horses (Harlow et al., 2013). These data suggested that antibiotic administration could disrupt the balance of the gastrointestinal microbiota and allow the proliferation of pathogenic bacteria.

Currently, there are no consensual and clear definitions or specifications regarding the concepts of both eubiosis and dysbiosis (Brüssow, 2020). However, Petersen and Round (2014) have defined dysbiosis as “any change in composition of resident commensal communities relative to the community found in healthy individuals.” According to Petersen and Round (2014) and Levy et al. (2017), dysbiosis results in a loss of commensals, a bloom of pathobionts, or a loss of diversity. In humans, common features of antibiotic-associated dysbiosis consist in modifications of the structure of the intestinal microbiota characterized by reduced diversity, a loss of key taxa, metabolic shift, and reduced resistance to pathogenic colonization (Lange et al., 2016). In horses, the paucity of studies on the effects of antibiotics on hindgut microbiota has not provided a complete picture of dysbiosis. However, high-throughput sequencing analysis revealed that orally administered TMS and metronidazole induced significant changes of bacterial structure and community membership after the cessation of antibiotic administration (Costa et al., 2015; Arnold et al., 2020). The major changes reported in both studies were a decrease in bacterial richness and diversity and a reduction of Verrucomicrobia abundance in the feces (Costa et al., 2015; Arnold et al., 2020). Whether the communities involved in fiber degradation were affected was not addressed. For better understanding how antibiotics shape the intestinal microbial diversity and structure, how these modifications might lead to ecosystem functioning or dysfunctions, with a special focus on fibrolysis, requires considering the microbial ecosystem as a whole.

In mammals, antibiotic-induced dysbiosis compromises immune homeostasis and has been linked to disorders involving inflammation and autoimmunity (Francino, 2016). It could promote the development of inflammatory gastrointestinal diseases (Lange et al., 2016). However, the horse’s immune response to variations in microbiota has been poorly investigated. Recently, the abundance of Verrucomicrobia and specific Clostridiales members has been positively correlated with increased expression levels of interleukin (IL)-10 and regulatory T-cell transcription factor Foxp3 on *post mortem* equine intestinal samples using high-throughput sequencing analysis and on the expression of several cytokine genes in the horse (Lindenberg et al., 2019). Therefore, it is important to clarify the potential relation between antibiotic-induced changes in hindgut microbiota and equine immunity for ensuring the animal’s health.

In their recent “News and Views” article, Yoo and Byndloss (2020) highlighted the need to answer to the questions “who is there?” and “what are they doing?” about the microbial community during dysbiosis and intestinal inflammation. Therefore, the main aim of this study was to use a general and comprehensive approach based on genotypic, functional, and environmental approaches for characterizing the hindgut ecosystem in *in vivo* response to oral TMS administration in horses. The effects of microbial ecosystem modifications, particularly on the cellulolytic bacteria and fibrolytic activity, on the host’s immune response and health were also assessed.

A probiotic blend composed of *Bifidobacterium* and *Lactobacillus* strains has been reported to contribute to the individual recovery from antibiotic therapy in a mouse model by reshaping and repopulating the microbiome (Grazul et al., 2016). These two lactic acid genera have also been shown to restore homeostasis to the intestinal barrier and to correct the balance of local and systemic immunological responses in experimental rodent models (Andrade et al., 2015). In horses, a preliminary study showed that supplementation with *Lacticaseibacillus rhamnosus* (formerly *Lactobacillus rhamnosus GG*; Zheng et al., 2020) given twice per day during a TMS challenge helped avoid the decrease in fecal cellulolytic counts observed in non-supplemented horses (Pyles et al., 2017). Here, dietary supplementation with *Bifidobacterium lactis*, *Lactobacillus acidophilus*, and *Ligilactobacillus salivarius* (formerly *Lactobacillus salivarius*; Zheng et al., 2020) was compared with placebo treatment to assess effects on the hindgut ecosystem, especially cellulolytic activity, and the equine immune balance after oral TMS administration.

## Materials and Methods

### Experimental Design, Animal Management, and Sampling Strategy

**FIGURE 1:**
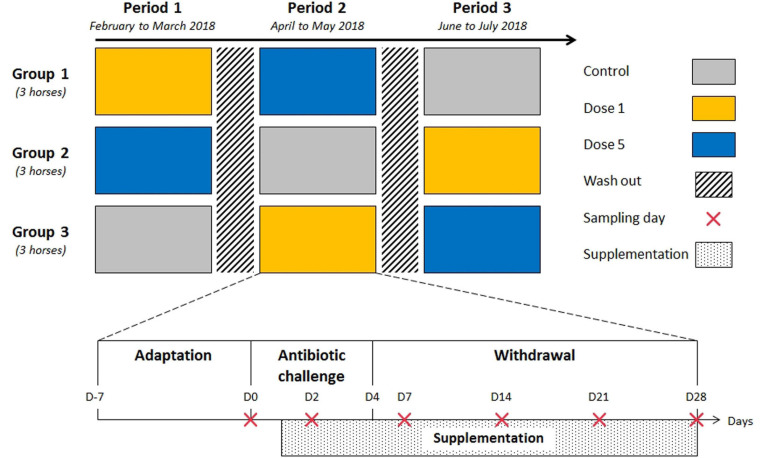
Outline of the trial design and experimental periods. Each experimental period comprised sample collection and the administration of a feed supplement. Nine adult horses were assigned randomly to one of the three feed supplement modalities for each 5-week period in a 3 × 3 Latin square design. Each period was separated by a 21-day washout period.

The protocol was approved by the local Committee on the Ethics of Animal Experiments of Grand Campus Dijon. Nine healthy adult Trotteurs Français geldings were used in the study. Their diet was formulated to supply 1.88% of body weight (BW) in the form of dry matter (DM) which exceeds the lowest recommended limit of daily forage intake (Harris et al., 2016), and 0.05% BW starch per meal, which is lower than the maximum recommendation for hindgut ecosystem health (Julliand et al., 2006; Supplementary Table 1). Horses were allocated randomly to three equal groups in terms of age, weight, and body condition score, then each group was enrolled in a 3 × 3 Latin square design composed of three experimental periods. Each experimental period comprised an adaptation phase to the diet and to the experimental routine (1 week, day [D]–7 to D–1), an antibiotic challenge phase (5 days, D0 to D4), and a withdrawal phase (3 weeks, D5 to D28: Figure 1). The three experimental periods were separated by 3-week washout periods during which horses received hay *ad libitum*. The antibiotic challenge consisted of 5 days of 30 mg/kg BW of TMS oral paste administration once daily. During each experimental period, horses received either dose 1 or dose 5 of a feed supplement, or a placebo (control) each morning before the meal. The two feed supplementations consisted of 3 *g* or 15 *g* doses of Floréquilibre CVX^®^ (Wamine and PileJe Laboratories, Paris, France) containing 4 × 10^10^ or 20 × 10^10^ colony-forming units (CFU) of *L. acidophilus* (LA201), *L. salivarius* (LA302), and *B. lactis* (LA304) blend per dose, respectively. These three strains are resistant to TMS.

Fresh feces and blood were sampled on all horses at days 0 (before the first TMS administration), 2, 7, 14, 21, and 28 by rectal grab and venipuncture of the jugular vein, respectively. Fecal samples were collected into sterile flasks filled to the top and held at 38°C during transportation until inoculation under a continuous flow of CO_2_ to maintain strict anaerobic conditions for microbiological analysis. Fecal samples were also subsampled sterilely and stored frozen for molecular biology analysis (–80°C). One gram of each fecal sample was dried at 65°C for 72 h to estimate the fecal DM. Then, feces were filtered, and the pH of the fluid portion was measured. The fecal filtrates were aliquoted with or without preservative solution composed of 4.25% H_3_PO_4_ and 1.0% HgCl^2^ and frozen (–20°C) for later analyses of VFAs and lactic acid, respectively. A final fecal subsample was suspended in 10 × stock phosphate-buffered saline (PBS) solution, centrifuged (1,500 *g* for 20 min at 4°C), and frozen (–20°C) for secretory immunoglobulin A (SIgA) analysis. Serum was obtained after a 2 h coagulation at room temperature and centrifugation of the blood samples (1,700 *g* for 20 min at 4°C). Serum samples were aliquoted and stored frozen for later immunoglobulin A (IgA) and cytokine analyses (–20°C).

### Bacterial 16S Ribosomal RNA Gene Sequencing Analysis

Total DNA was extracted from 0.25 *g* fecal content as described by Yu and Morrison (2004). The quantity and purity of obtained DNA were assessed on a spectrophotometer (Eppendorf, Hamburg, Germany).

Amplification of the V3–V4 hypervariable region of the bacterial 16S ribosomal RNA (rRNA) gene, sequencing, and bioinformatics analysis were performed as described by Grimm et al. (2020). Briefly, after amplification by a first round of polymerase chain reaction (PCR) amplification, the DNA samples were electrophoresed on 2% agarose gels to verify the amplification. Then, 16S rRNA gene amplicons of each sample were sent to a specialized platform to perform the sequencing analysis (Genotoul Bioinformatics Platform, http://bioinfo.genotoul.fr/, Toulouse, France). Amplicons were purified and submitted to a second round of PCR, and the resulting PCR products were sequenced using an Illumina MiSeq run of 250 base-paired ends according to the manufacturer’s instructions (Illumina Inc., San Diego, CA, United States). Bioinformatics analyses were performed using the FROGS pipeline (Find Rapidly OTU with Galaxy Solution; http://frogs.toulouse.inra.fr). Resulting Operational Taxonomic Units (OTUs) that were not present in at least two samples or whose abundance was <5 × 10^–5^ were removed. Remaining OTUs where then aligned to the SILVA123 16S database (Quast et al., 2013; Yilmaz et al., 2014) using BLAST. Relative abundances of the different families and genera were calculated and expressed as the percent of the total number of OTUs per sample. To estimate the diversity of the microbial community, within-sample Shannon and Simpson indexes were calculated from the abundance table using FROGS. All raw sequences obtained during this study were submitted to the NCBI Sequence Read Archive (accession number PRJNA703761).

### *Clostridium difficile* Quantification

Quantitative real-time PCR (qPCR) was performed to measure *C. difficile* in fecal DNA extracts as described by Bandelj et al. (2013). Oligonucleotide primers CDIFF16S-F (5’TTGAGCGATTTACTTCGGTAAAGA3’) and CDIFF16S-R (5’TGTACTGGCTCACCTTTGATATTCA3’), and CDIFF16S TaqMan probe (FAM-CCACGCGTTACTCACCCGTCCG; MGW, Eurofins Genomics, Germany) specific to *C. difficile* 16S rRNA gene (NCBI accession number AB548672; https://www.ncbi.nlm.nih.gov/nuccore/AB548672) were used for the qPCR assay (Penders et al., 2005). The qPCR mixture (final volume 25 μL) was composed of FastStart Universal Probe Master Mix 2X (12.5 μL; Merck KGaA, Darmstadt, Germany), primers (900 nM), probe (200 nM), and DNA (1 μL). Twenty nanograms of bovine serum albumin (BSA) were added to the mixture to reduce inhibition. The qPCR runs were performed in a 96-well plate format using CFX96 Real-Time System (Thermo Fisher Scientific, Indianapolis, IN, United States). Thermal cycling conditions used were as detailed by Bandelj et al. (2013). One negative control was included to verify the absence of any contamination. An internal positive standard was used and consisted in 1 ng/μL of *C. difficile* ATCC 9689 (DSMZ, Leibniz Institute, Germany). Results are expressed in cells of *C. difficile* cells per mL, based on a standard curve obtained from serial dilution of the positive standard. The limit of quantification was 2.25 × 10^5^ cells/mL. The slopes of the calibration lines were between –3.02 and –3.25 (*r*^2^ = 0.99).

### Bacterial Functional Group Analysis

Enumeration from fecal samples was performed using conventional anaerobic culture techniques. To maintain strict anaerobic conditions, fresh fecal samples were diluted in a mineral solution under continuous flow of CO_2_ (Bryant and Burkey, 1953). Total anaerobic, starch-utilizing, and lactic acid-utilizing bacteria were inoculated on non-selective medium and on selective media containing starch and lactic acid, respectively (Grimm et al., 2020), in roll tubes (Hungate and Macy, 1973), and incubated for 48 h at 38°C. Cellulolytic bacteria were cultured anaerobically in roll tubes containing a complex liquid medium with one filter paper strip as cellulose source for 14 days at 38°C (Halliwell and Bryant, 1963). The inoculated dilutions represented 10^–5^ to 10^–7^ mL of fecal contents for all media. Bacterial concentrations were determined by colony count (CFU) or using the method of the most probable Mc Grady number, solely for cellulolytic bacteria (Halliwell and Bryant, 1963). Finally, all bacteria concentrations were converted into base 10 logarithms.

### Determination of Fermentation End Products

Total VFA, acetic (C2), propionic (C3), iso-butyric (iC4), butyric (C4), iso-valeric (iC5), and valeric (C5) acid concentrations were assayed by gas–liquid chromatography (Clarus 500; PerkinElmer, Courtaboeuf, France; Jouany, 1982). Ratio [(C2 + C4)/C3] was calculated according to Sauvant et al. (1994). D- and L-lactic acid concentrations were measured spectrophotometrically at 340 nm (MRX Revelation Microplate Reader, Dynatech Laboratories. Guyancourt, France) using enzymatic colorimetric rapid) assay kits (Megazyme International Ireland Ltd, Wicklow, Ireland) as described by Grimm et al. (2017). Results are expressed in mmol/L.

### Immunoglobulin A Concentration Analysis

Seric IgA and fecal SIgA were quantified in duplicate using enzyme-linked immunosorbent assays and completed with the manufacturer’s accessory kits (Horse IgA Quantification Set, Bethyl Laboratories Ltd., Montgomery, TX, United States), which were slightly modified in our laboratory. Samples were centrifuged at 5,000 *g* for 5 min. The horseradish peroxidase dilution was fixed at 1/250,000. Absorbance was read at 450 nm using a spectrophotometer (MRX Revelation Microplate Reader). Results are expressed as the mean mg/mL of serum IgA and mean μg/g DM SIgA.

### Cytokine Profile Analysis

A commercially available equine-specific multiplexed cytokine/chemokine magnetic bead kit (EMD Millipore, Merck, Darmstadt, Germany) was used to simultaneously test for IL-6, IL-10, and TNF-α in serum samples using a 96-well plate format and following the manufacturer’s instructions. All samples were run without dilution. Plates were read using Bio-Plex Multiplex System instrumentation (Bio-Rad Laboratories, Hercules, CA, United States) using Luminex xMAP technology (Luminex Corporation, Austin, TX, United States). A minimum bead count of 50 for each cytokine/chemokine was acquired for data analysis. Data were analyzed using Bio-Plex Manager Software 6.1 (Bio-Rad Laboratories, Hercules, CA, United States). Results are expressed in pg/mL of serum. Concentrations that were out of ranges were replaced by the detection limit values for IL-6 (14.50 pg/mL), IL-10 (45.42 pg/mL), or TNF-α (2.99 pg/mL).

### Statistical Analyses

Statistical analysis was performed using the MIXED procedure in SAS software version 9.3 (SAS Institute Inc., Cary, NC, United States) with a model including the period, supplementation type, day, and the interaction between supplementation type and day as fixed effects. The model included “horse” as a random effect and “days” as repeated measures. The period was used as an intercept for each horse to avoid individual variations along the duration of the experimental trial. Thus, only the effects of supplementation, day, and their interaction will be discussed here. In case of significant day × supplementation interactions, means were compared using the pdiff option in the LSMeans statement. The significance threshold was set at *P* ≤ 0.05, and trends were considered at *P* ≤ 0.10.

Partial least squares discriminant analysis (PLS-DA) was performed with SIMCA 15 (Umetrics, Umea, Sweden) on the genus-abundance table to discriminate both the effect of the day and the effect of supplementation modalities for each day. The significance of PLS-DA models was verified with RX and RY parameters, indicating the fitness and prediction accuracy of the model at *P* ≤ 0.05. PLS-DA models were used to extract the most discriminative genera for each day. Discriminative genera with variable importance in projection (VIP) values > 2 and *P* ≤ 0.05 (using multiple-sample analysis of variance tests) were considered as relevant.

Linear discriminant analysis effect size (LEfSe) was performed with the Galaxy software package of the Huttenhower Laboratory (Segata et al., 2011) to determine significant taxonomic differences between the supplementations. This method employs the factorial Kruskal–Wallis sum-rank test (α = 0.05) to identify taxa with significant differential abundances between modalities (using all-against-all comparisons), followed by linear discriminant analysis (LDA) to estimate the effect size of each differentially abundant feature based on a threshold of 3 log LDA scores. Significant taxa were used to generate a taxonomic cladogram illustrating differences between supplementation modalities.

## Results

### Clinical Follow-Up of Horses

At the beginning of the first washout period, one horse developed a laryngopharyngitis and was given Duplocilline^®^ (33 mL, IM) for 3 days. This horse recovered well and quickly and was kept in the experimental design. Some clinical signs of colic were noticed in another horse on D16 during the first experimental period. This horse received one injection of Estocelan^®^ (20 mL, IV). The diet was not changed. The horse recovered immediately, and the experiment was continued. All other horses were healthy during the entire trial. They did not develop diarrhea, but loose feces were observed at D2. Horses had stable body weight and condition over the full experimental design.

### Modifications of Fecal Bacterial Diversity, Composition, and Structure

After quality control, 2,563,477 reads of V3–V4 16S rRNA sequences were obtained from the 162 samples with 15,824 ± 7,029 sequence reads per sample. In all, 1,996 OTUs were obtained after clustering the sequences. Sequences were assigned to 9 phyla, 15 classes, and 17 orders (Supplementary Figure 1). Firmicutes was the dominant phylum (50.74% ± 4.63) followed by Bacteroidetes (40.87% ± 4.06), Proteobacteria (4.38% ± 1.52), and Fibrobacteres (2.46% ± 1.72). At a lower taxonomic level, 28.15% of the total relative abundance included unknown genera. No day × supplementation interaction or supplementation effect was observed for fecal bacterial richness and diversity. The latter was significantly lower at D2 compared with D0 (Table 1).

**TABLE 1:** Variation in fecal bacterial richness and diversity according to the day in horses subjected to anoral challenge with TMS from D0 to D4.

Sampling day	Number of observed OTU	Shannon index	Simpson index	InvSimpson index
D0*	1,146^ab^	5.95^a^	0.9929^a^	150^a^
D2	1,087^b^	5.73^b^	0.9908^b^	121^c^
D7	1,183^a^	5.85^a^	0.9918^ab^	128^bc^
D14	1,163^ab^	5.86^a^	0.9915^ab^	136^abc^
D21	1,210^a^	5.95^a^	0.9926^a^	155^a^
D28	1,231^a^	5.92^a^	0.9921^a^	145^ab^
Standard deviation	182	0.25	0.0030	30
*P*-value				
Day	**0.0253**	**0.0004**	**0.0176**	**0.0029**
Supplementation	0.8175	0.6688	0.3549	0.3947
Day * Supplementation	0.9158	0.9331	0.8913	0.7668

*D0: Basal value before the first TMS administration.

*For each column, means with different superscripts differ at *P* < 0.05.*

*Significant *P*-values are in bold.*

The relative abundance of the Fibrobacteres phylum decreased from 2.83% at D0 to 1.78% at D2 (*P* = 0.0242) and returned to its basal state at D7. The relative abundance of the Tenericutes phylum increased from D0 (0.07%) to D2 (0.24%; *P* = 0.0434) and returned to its basal range at D7. Modifications were also highlighted in the feces of horses at the family (Supplementary Table 2) and genus (Supplementary Table 3) levels according to day. Among the 39 families and the 99 genera observed, 20 and 39 (representing 74.95% and 46.18% of the total family and genus relative abundances, respectively) showed differences related to the day.

**FIGURE 2:**
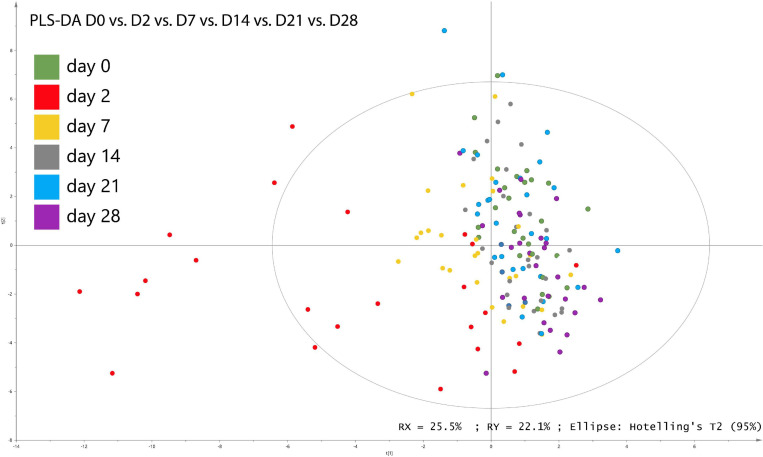
Partial least square discriminant analysis (PLS-DA) of bacterial genera associated with days of sampling in fecal samples from horses subjected to TMS oral challenge from D0 to D4.

Using PLS-DA treatments of the genomic dataset of fecal samples, differences between the relative abundances of fecal bacterial populations were reported for each day at the genus level (Figure 2). Regardless of supplementation, the PLS-DA score plots revealed clear disparities in the genomic datasets and highlighted clustering based on D2 in bacterial community structure along the first and second X-variate axes. *[Eubacterium] ruminantium group, Christensenellaceae R-7 group, Lachnoclostridium*, *Lachnospiraceae UCG-003*, *Mailhella*, *Noviherbaspirillum*, and *Ruminococcaceae UCG-010* were identified as discriminant genera (VIP > 0.2 and *P* ≤ 0.05).

Awe found a day × supplementation interaction for the relative abundance of *Treponema 2* genus and for upper taxonomic levels (Spirochetes > Spirochetes > Spirochaetales > Spirochaetaceae; *P* = 0.0058) at D7 and D14 (Supplementary Figure 3A). The relative abundances of the *Anaerovibrio* genus and the higher taxonomic levels (Negativicutes > Selenomonadales > Veillonellaceae) were greater in the feces of horses supplemented with Dose 5 (*P* = 0.0012; Supplementary Figure 2B). At the genus level, the relative abundance of *Lachnospiraceae UGC 008* was higher in the feces of control horses than in the supplemented groups (*P* = 0.0076) whereas the relative abundance of *Ruminococcaceae UGC 013* was higher in the fecal content of control horses than in horses receiving Dose 5 only (*P* = 0.0023; Supplementary Figure 2B).

Key bacterial groups that most likely explained the differences between the feces of control and supplemented animals were identified using the LEfSe analysis. A circular cladogram was generated to show the differentially abundant taxa (Supplementary Figure 3) and LDA coupled with the effect size measurements identified bacterial genera that were most likely responsible for the different bacterial microbiota composition depending on the supplementation modalities (Table 2). Considering the LEfSe results, OTUs were selected and were associated with each supplementation group in the fecal samples. At the genus level, the relative abundances of *Desulfovibrio*, *[Eubacterium] nodatum group*, and *Family XIII AD3011 group* were enriched in the fecal microbiota of the control horses. The relative abundances of *Eubacterium*, *Marvinbryantia*, *Lachnospiraceae AC2044 group*, *Rikenellaceae RC9* gut group, and *Treponema 2* were associated with the fecal microbiota of horses supplemented with Dose 1. The fecal microbiota of horses supplemented with Dose 5 was enriched with *Prevotellaceae UCG-003* and *Subdoligranulum*.

**TABLE 2:** LDA scores and mean relative abundance of the discriminant bacterial genera enriched in the fecal microbiota of horses under different supplementation modalities.

Bacterial genera	Mean ± S.D.	LDA score	*P*-value
**Control**			
*Desulfovibrio* (Proteobacteria > Deltaproteobacteria > Desulfovibrionales > Desulfovibrionaceae)	0.15 ± 0.07	3.28	0.0349
*[Eubacterium] nodatum group* (Firmicutes > Clostridia > Clostridiales > Family XIII)	0.07 ± 0.05	3.23	0.0193
*Family XIII AD3011 group* (Firmicutes > Clostridia > Clostridiales > Family XIII)	0.29 ± 0.17	3.08	0.0028
**Dose 1**			
*Treponema 2* (Spirochetes > Spirochaetia > Spirochaetales > Spirochaetaceae)	4.34 ± 1.29	3.62	0.0176
*Rikenellaceae RC9 gut group* (Bacteroidetes > Bacteroidia > Bacteroidales > Rikenellaceae)	5.54 ± 2.16	3.28	0.0448
*Eubacterium* (Firmicutes > Clostridia > Clostridiales > Eubacteriaceae)	0.03 ± 0.02	3.03	0.0465
*Marvinbryantia* (Firmicutes > Clostridia > Clostridiales > Lachnospiraceae)	0.36 ± 0.20	3.02	0.0063
*Lachnospiraceae AC2044 group* (Firmicutes > Clostridia > Clostridiales > Lachnospiraceae)	4.57 ± 1.61	3.01	0.0100
**Dose 5**			
*Subdoligranulum* (Firmicutes > Clostridia > Clostridiales > Ruminococcaceae)	0.06 ± 0.04	3.42	0.0345
*Prevotellaceae UCG-003* (Bacteroidetes > Bacteroidia > Bacteroidales > Prevotellaceae)	2.30 ± 0.76	3.25	0.0062

*Only genera meeting a significant LDA threshold > 3 and *P* < 0.05 are shown.*

The concentrations of *C. difficile* cells were lower than the limit of quantification of 2.25 × 10^5^ cells/μL in all samples.

### Modifications of the Fecal Bacterial Functional Groups and Fermentation End Products

**FIGURE 3:**
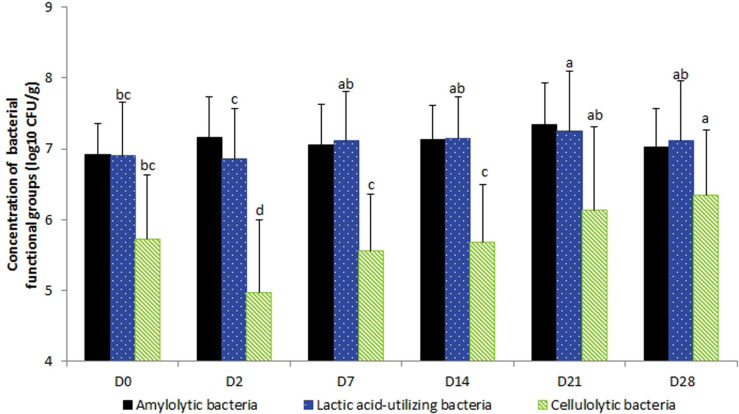
Variations in the bacterial functional group concentrations according to the day in fecal samples of horses subjected to TMS oral challenge from D0 to D4. For each group, mean concentrations with different superscripts differ at *P* < 0.05. ^∗^D0: Basal value before the first TMS administration.

No day × supplementation effect was found. The concentrations of fecal bacterial functional groups did not differ significantly according to supplementation. From D2 to D7, lactic acid-utilizing bacterial concentrations increased (*P* = 0.0153). The concentration of cellulolytic bacterial concentrations decreased from D0 to D2 (*P* < 0.0001) and then increased progressively after the end of the experimental period (Figure 3). Total anaerobic and starch-utilizing bacterial concentrations remained stable during the 28-day experimental period.

**FIGURE 4:**
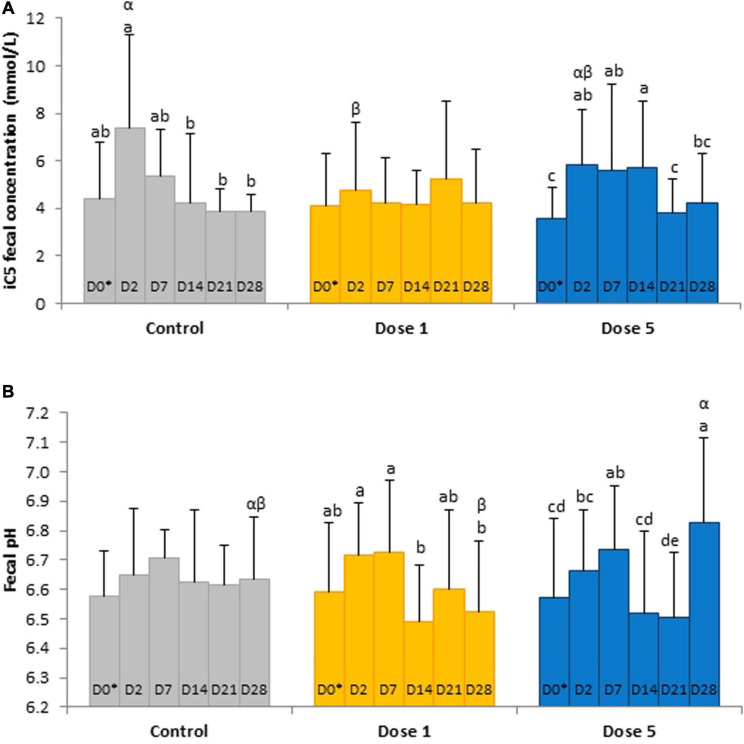
Depiction of significant day × supplementation interactions regarding **(A)** isovaleric acid concentrations and **(B)** pH in fecal samples from horses subjected to an oral challenge with TMS from D0 to D4 by day. Different Latin letter superscripts show significant variations between days for each supplementation. Different Greek letter superscripts indicate significant variations between supplementations for each day. ^∗^D0: Basal value before the first TMS administration.

Fecal acetic, iso-butyric, and valeric acid concentrations and the VFA ratio decreased significantly from D0 to D2 whereas total VFA concentrations decreased significantly from D14 to D21 (Table 3). A day × supplementation effect was found for fecal isovaleric acid concentration (*P* = 0.0244). At D2, the isovaleric acid concentration was lower in the fecal samples of horses supplemented with Dose 1 than in the fecal contents of controls (*P* = 0.0296). The fecal isovaleric acid concentrations of Dose 5 (*P* = 0.0123) and control (*P* = 0.0445) groups varied according to day whereas they stayed constant for horses supplemented with Dose 1 (Figure 4A).

**TABLE 3:** Variations in bacterial fermentation end-product concentrations (mmol/L) and ratios reported in fecal samples of horses subjected to an oral challenge with TMS from D0 to D4 by day.

Concentrations of fecal bacterial fermentation end product (mmol/L)	D0*	D2	D7	D14	D21	D28	Mean ± S.D.	Day *P*-value	Supplementation *P*-value	Interaction *P*-value
**Total lactic acid**	1.20	1.21	1.28	1.41	0.89	1.15	1.19 ± 0.66	0.0896	0.3262	0.9124
**D-lactic acid**	0.64	0.65	0.68	0.75	0.49	0.61	0.64 ± 0.35	0.1236	0.3246	0.9399
**L-lactic acid**	0.55	0.56	0.60	0.66	0.40	0.54	0.55 ± 0.31	0.0674	0.3282	0.8648
**Lactic acid ratio** *(L-lactic acid/D-lactic acid)*	0.86	0.84	0.90	0.89	0.81	0.92	0.87 ± 0.19	0.0904	0.5862	0.9323
**Total VFAs**	57.85^ab^	64.58^a^	62.00^ab^	64.76^a^	55.97^b^	56.20^b^	60.23 ± 17.38	**0.0251**	0.2061	0.5330
**Acetic acid** *(C2)*	40.88^bc^	46.40^a^	44.24^abc^	45.48^ab^	39.21^c^	39.78^c^	42.66 ± 12.37	**0.0168**	0.2400	0.4690
**Propionic acid** *(C3)*	11.39	11.70	11.77	12.92	11.12	10.94	11.64 ± 3.52	0.0956	0.1132	0.6380
**Iso-butyric acid** *(iC4)*	0.86^b^	1.08^a^	1.02^a^	0.96^ab^	0.88^b^	0.87^b^	0.94 ± 0.33	**0.0012**	0.5587	0.1100
**Butyric acid** *(C4)*	3.36	3.62	3.42	3.85	3.37	3.30	3.49 ± 1.29	0.1912	0.3349	0.5799
**Iso-valeric acid** *(iC5)*	0.98	1.27	1.11	1.09	0.99	0.95	1.06 ± 0.55	0.0526	0.7213	**0.0244**
**Valeric acid** *(C5)*	0.39^b^	0.52^a^	0.45^ab^	0.45^a^	0.40^b^	0.37^b^	0.43 ± 0.23	**0.0109**	0.6320	0.0787
**VFA Ratio** (*C2* + *C4*)*/C3*	3.94^b^	4.51^a^	4.05^b^	3.85^b^	3.95^b^	3.97^b^	4.04 ± 0.78	**0.0133**	0.3112	0.3171

* D0: Basal value before the first TMS administration.

*Means with different superscripts differ at *P* < 0.05.*

*Significant *P*-values are in bold.*

**FIGURE 5:**
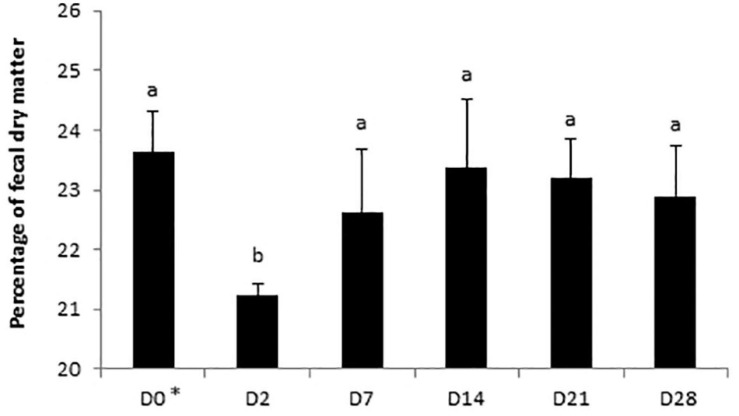
Variations of the percentage of dry matter in fecal samples of horses subjected to an oral challenge TMS from D0 to D4 by day. Means with different superscripts differ significantly at *P* < 0.05. ^∗^D0: Basal value before the first TMS administration.

A day × supplementation interaction was reported for the fecal pH of horses (*P* = 0.0240; Figure 4B). The pH of both Dose 1 (*P* = 0.0166) and Dose 5 (*P* = 0.0007) groups varied according to the day whereas the pH of the control group was not affected by the day. At D28, the fecal pH of horses supplemented with Dose 5 was higher than those of both control horses and horses supplemented with Dose 1 (*P* = 0.0024). A decrease of the fecal DM was observed at D2 (*P* = 0.0035; Figure 5).

### Modifications of the Equine Immune System Response

**FIGURE 6:**
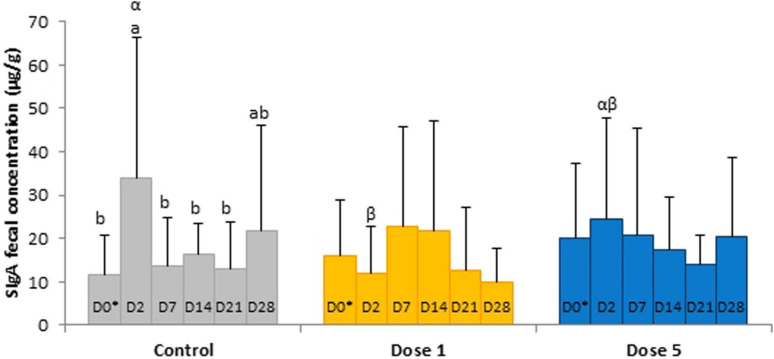
Depiction of significant day × supplementation interactions regarding SIgA concentrations in fecal samples of horses subjected to anoral challenge with TMS from D0 to D4. Different Latin letter superscripts show significant variations between days for each supplementation. Different Greek letter superscripts indicate significant variations between supplementations for each day. ^∗^D0: Basal value before the first TMS administration.

**FIGURE 7:**
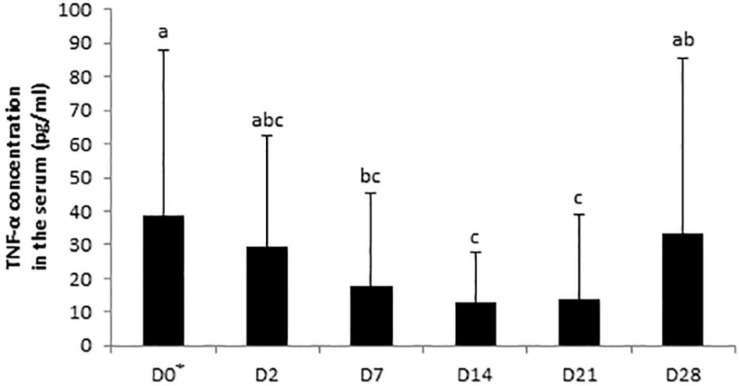
Variations in TNF-α concentrations in serum samples of horses subjected to an oral challenge with TMS from D0 to D4 by day. Means with different superscripts differ at *P* < 0.05. ^∗^D0: Basal value before the first TMS administration.

No significant day × supplementation interaction, day, and supplementation effects were found for IgA concentrations in the serum samples. A tendency was observed for the fecal SIgA concentration in the day × supplementation interaction (*P* = 0.0780). At D2, the fecal concentration of SIgA of control horses (34.04 ± 32.31 μg/g) was higher than the group supplemented with Dose 1 (12.08 ± 10.56 μg/g; *P* = 0.0166). SIgA concentrations varied according to the day in the feces of control horses (*P* = 0.0408) whereas no significant modifications were reported in both supplemented groups (Figure 6). For the analysis of IL-6, IL-10, and TNF-α, 158, 125, and 58 out of 162 samples were under the limit of detection, respectively. An effect of day was observed for the TNF-α concentration which decreased from D0 to D7 (*P* = 0.0214; Figure 7).

## Discussion

In this study, to understand how antibiotics affect the overall equine hindgut ecosystem, specific attention was paid to the holistic and comprehensive analysis of both the member composition and the metabolic state of the bacterial communities. This enabled us to observed genotypic, functional, and environmental dimensions of the equine microbial hindgut ecosystem following oral TMS administration.

In our experimental conditions, TMS induced dysbiosis as defined by Petersen and Round (2014): a loss of diversity, a bloom of potential pathobionts, and a loss of commensals. A significant loss of the equine fecal bacterial diversity was observed from the second day of oral administration of TMS. Previous observations reported a decrease in bacterial diversity at the end of TMS administration (Costa et al., 2015). In fact, our data indicated that alteration of the bacterial diversity occurred rapidly after the first administration of TMS. We expected an increase in bacterial pathogens such as *C. difficile* and *Salmonella* spp., as previously reported during TMS challenge using culture-dependent methods (Harlow et al., 2013). However, our investigation of *C. difficile*, using a qPCR method, did not show the presence of this pathogen in the fecal samples. In healthy adult horses, the carriage prevalence of *C. difficile* in the gastrointestinal tract usually ranges between 0% and 10% (Diab et al., 2013). Therefore, the first hypothesis was that the nine horses included in the present study did not carry *C. difficile* during this experimental period. However, the limit of quantification of our qPCR method was 2.25 × 10^5^ cells/mL which is higher than the viable number of *C. difficile* found by Harlow et al. (2013). qPCR investigation probably lacked sensitivity to detect *C. difficile* variations in feces if it had occurred. In contrast, the relative abundance of *Alloprevotella*, *Desulfovibrio*, and *Prevotella* increased during TMS administration. Regarding their potential detrimental effects on the host health, several species of *Alloprevotella* (Liew et al., 2019), *Prevotella* (Larsen, 2017), and *Desulfovibrio* (Rowan et al., 2010) could be considered as pathobionts. We hypothesize that the bloom of such opportunistic bacteria could be explained by the disappearance of colonization resistance induced by the loss of commensal members (Lawley and Alan, 2012). In fact, here a loss of commensals was observed along with the loss of bacterial diversity and the bloom of potential pathobionts.

The relative abundance of *[Eubacterium] ruminantium group*, *Lachnospiraceae AC2044 group*, *Ruminococcaceae UCG-014*, and *Saccharofermentans* decreased on the second day of TMS treatment. These genera belong to the Lachnospiraceae and Ruminococcaceae families, which are supposed to play a role in plant wall degradation and fermentation thanks to their fibrolytic activity (Biddle et al., 2013). Moreover, the relative abundance of Fibrobacter, another main fiber degrader (Ransom-Jones et al., 2012), also declined at the same time. These genomic results were supported by the significant fall in cellulolytic bacterial concentration observed using culture-dependent technique. This was consistent with a previous study using oral treatment with TMS (Harlow et al., 2013). In the horse, the disappearance of such keystone bacteria in the hindgut might be detrimental for the host’s digestive function and health (Julliand and Grimm, 2016). Based on PLS-DA analysis, *Lachnoclostridium* (Lachnospiraceae) and *Ruminococcaceae UCG-010* (Ruminococcaceae) appeared to be two discriminant genera whose relative abundance increased during TMS administration. This shift in potential fibrolytic members might reflect an adaptive capacity and strategy of the microbiota to ensure the host’s fiber-digesting function and health. The VFA ratio as defined above is a relevant marker of fibrolytic activity (Grimm et al., 2017) and normally ranges from 4.3 to 6.0 in the hindgut of horses fed a high-fiber diet (Julliand and Grimm, 2017). It decreased significantly in the hindgut when horses nourished high-starch diet demonstrating a decrease in microbial fibrolytic activity. On the contrary, no modification was found in the feces when the diet was changed from high fiber to high starch (Grimm et al., 2020). In the present trial, although horses were fed a high-fiber diet, the VFA ratio was greater than 4.3 only 2 days after the start of TMS administration because of the increase in fecal acetic acid concentration. Nevertheless, our genomic and microbiologic results did not indicate any increase in bacterial fibrolytic activity. Consequently, fecal VFA concentrations and ratios might not depict the microbial metabolic activity during TMS use, but rather an alteration in VFA metabolism and absorption rates.

The relative abundances of *[Eubacterium] ruminantium group* and *Mailhella* reduced from the second day of TMS administration whereas the relative abundance of *Christensenellaceae R7 group* was increased. These genera are known to be butyric acid producers with a positive impact on the host’s health (Perea et al., 2017; Huang et al., 2019). Given that we found no modification of butyric acid fecal concentration, these observations further suggest that butyric acid-producing genera might reorganize to guarantee the bacterial community and the host’s requirements. Butyric acid is a cellular mediator regulating multiple intestinal functions including the control of diarrhea (Bedford and Gong, 2018). Diarrhea is a serious adverse effect of antibiotic use in horses, and TMS administration has been associated with its occurrence (McGorum and Pirie, 2009, 2010). In humans, the main explanation for antibiotic-associated diarrhea onset is the reduction in VFA production which decreases colonic stimulation of fluid absorption and leads to an increase of water in the lumen of the large intestine (Binder, 2010). Here, no episode of diarrhea was observed but a transient decrease in fecal DM of all horses occurred 48 h after the first TMS administration in parallel with increases in acetic and valeric acid concentrations. The concentration of VFAs in feces represents the residual non-absorbed VFAs produced by microbial fermentation in the hindgut. An increase of fecal VFA concentrations might be the result of a lower rate of absorption across the equine colonic luminal membrane into colonocytes, which is monocarboxylate/H + symporter-dependent (Shirazi-Beechey, 2008). Thus, it is hypothesized that TMS treatment impaired the hindgut mucosa and its absorption mechanisms. Our *in vivo* equine study did not allow a direct histological observation of the hindgut mucosa for assessing potential alterations. An association has been demonstrated between the histopathological pattern of inflammation, cytokine expression profiles, and number of infiltrating regulatory T cells in rectal biopsies from horses affected with active chronic intestinal inflammation (Olofsson et al., 2015). This suggests that the mucosal immune system is affected when the hindgut mucosa is damaged. Therefore, we explored biomarkers in the feces, easily accessible biological samples, which indicated changes in the local innate immune system. We observed an increase in SIgA fecal concentrations in control horses after 2 days of oral administration of TMS reflecting an alteration in the mucosal immune response, similarly to data reported in murine models treated orally with broad-spectrum antibiotics (Aguilera et al., 2015). In adult mammals, the intestinal microbiota induces the maturation of the immune system, and in turn, the amount and quality of SIgA directly influence the diversity and phylogenetic structure of bacterial communities (Kawamoto et al., 2014). Recent evidence has also linked alterations of the systemic immune systems to the microbiota, but the implicated mechanisms—direct or indirect—remain mostly unknown (Belkaid and Harrison, 2017). In this study, only concentrations of the early proinflammatory cytokine TNF-α could be interpreted, as most of IL-6 and IL-10 values were under the range of detection. We expected an increase in TNF-α concentration in the serum as a consequence of the local enhancement of TNF-α expression in colonic tissues which was reported in vancomycin and polymyxin B-induced dysbiotic mice (Ran et al., 2020). Nevertheless, our results displayed a progressive decrease in TNF-α serum concentration in association with TMS-induced dysbiosis in all horses. After 7 days of antibiotic administration, the blood mononuclear cells of healthy human subjects demonstrated a reduced *in vitro* capacity to release TNF-α after lipopolysaccharide stimulation. The authors concluded that systemic innate immune defenses could be affected during an antibiotic challenge in response to microbiota disruption (Lankelma et al., 2016). In the same way, the change we observed in TNF-α in the serum of horses might be related to the modification of the systemic immune response during antibiotic-induced dysbiosis. Further investigations are required to evaluate these results.

Another aim of this study was to investigate the effect of two doses of a blend of *L. salivarium*, *L. acidophilus*, and *B. lactis* on the hindgut ecosystem, especially cellulolytic activity, and immune balance of horses after an oral administration of TMS. Supplementing horses for maintaining the microbiota in a state more resistant to disturbances could be a way to delay or prevent the development of microbiota-related diseases. Few studies have used an antibiotic challenge to disturb the intestinal microbiota and evaluate the impact of probiotic strain on this perturbation (Pyles et al., 2017). In our study, the supplemented horses displayed a similar antibiotic-induced dysbiosis as observed in control horses, suggesting that the use of this probiotic blend did not maintain the hindgut microbiota of the horses subjected to TMS administration in our conditions. Nor did we not observe maintenance of fibrolytic function during TMS administration. Only a few genera that could be implicated in structural carbohydrate degradation such as *Eubacterium* and *Rikenellaceae RC9 gut group* were enriched in the feces of horses receiving Dose 1 (Wade, 2006; Ren et al., 2020). This contrasted with another study indicating that in horses subject to TMS administration and receiving *L. rhamnosus* twice daily, cellulolytic bacteria remained at stable concentrations in the feces (Pyles et al., 2017). The decrease in fecal pH that we measured 9 days after the last TMS administration in supplemented horses might suggest a faster recovery of fibrolysis after the TMS challenge. However, this was not associated with any modification of fecal VFA concentrations or bacterial composition in the supplemented groups. Unfortunately, our results did not show an impact of feed supplementation on the fecal bacterial recovery following TMS treatment. Interestingly, *Marvinbryantia* and *Lachnospiraceae AC2044 group* or *Subdoligranulum* were enriched when horses were supplemented with Dose 1 or Dose 5, respectively. The enrichment in these butyric acid-related genera (Thomas et al., 2014; Jirsova et al., 2019; Yang et al., 2019) might help support mucosal integrity because of the anti-inflammatory effects of butyric acid (Furusawa et al., 2013). This hypothesis was reinforced by the fecal SIgA levels, which were maintained during antibiotic administration when horses were supplemented with Dose 1 or Dose 5. In humans, the same blend as the one we used (*L. salivarium*, *L. acidophilus*, and *B. lactis*) reduced inflammation 72 h after ciprofloxacin/metronidazole treatment in patients suffering from acute uncomplicated diverticulitis compared with non-supplemented patients (Petruzziello et al., 2019). Therefore, this probiotic blend might display similar anti-inflammatory properties in equines subject to antibiotic administration and the response might be dose related. In horses receiving Dose 1, lower fecal SIgA concentrations were found 2 days after the first TMS administration compared with control horses and horses supplemented with Dose 5. Similar results were observed regarding isovaleric acid fecal concentrations. In addition, when supplemented with Dose 1, horses exhibited a trend for higher fecal DM content (*P* = 0.0902). This might reflect a better colonic absorption of VFAs and water, and utilization of isovaleric acid in the hindgut with Dose 1 than Dose 5. Further studies are needed to understand the mechanism of action and the most relevant dose and application of the *L. salivarium*, *L. acidophilus*, and *B. lactis* blend in horses.

Here, we measured the capacity of the horse hindgut ecosystem to recover from the impact of TMS perturbation during a withdrawal period of 23 days. The fecal bacterial recovery to TMS oral administration had previously been investigated for 7 days after the treatment ended using culture-dependent methods (Harlow et al., 2013) to 25 days using culture-independent methods for characterizing the fecal microbiota (Costa et al., 2015). A previous study reported a recovery in bacterial richness and diversity in fecal samples 9 days after the cessation of TMS administration (Costa et al., 2015). We confirmed that bacterial diversity was back to its basal value 10 days after treatment cessation, and given that we sampled after 2 days of withdrawal, it appeared that the recovery occurred quickly. Similarly, after 2 days of withdrawal from TMS treatment, we found a rapid recovery of the bacterial structure and community membership with the persistence of few modifications in the genera present. This confirmed earlier results indicating that the changes induced by TMS tend not to last for more than 9 days after stopping treatment (Costa et al., 2015). The functional bacterial groups also returned to their basal value from 2 days after stopping treatment. This contrasts with a previous report indicating that cellulolytic bacterial concentrations were still very low after a withdrawal period of 7 days (Harlow et al., 2013). Differences in the duration during which horses received the TMS administration (5 days in our study instead of 7) could account for this variation in the persistence of cellulolytic bacteria depression. The longer recovery of cellulolytic bacteria observed by Harlow et al. (2013) could also be explained by differences in the hindgut microbiota composition before antibiotic perturbation and to the presence of *C. difficile*, which can inhabit the same ecological niche as cellulolytic bacteria. Interestingly, in the conditions of our study, cellulolytic bacterial counts increased progressively and peaked 24 days after the end of TMS treatment. Concomitantly, the relative abundances of *[Eubacterium] ruminantium group* and *Fibrobacter* were overrepresented 2 to 3 weeks after the end of TMS administration in comparison with the basal state. Such genera are involved in hindgut fibrolysis (Wade, 2006; Ransom-Jones et al., 2012). This recovery of the microbiome could be supported by enrichment in specific carbohydrate-degradation and energy-production pathways which are driven by recovery-associated bacteria (Chng et al., 2020). In equines, recovery-associated bacteria might help the rehabilitation of keystone bacteria responsible for the fibrolytic activity which is crucial for health. In this study, horses were continuously fed with a high-fiber diet and the antibiotic administration did not affect their dietary intakes. Giving horses a high-fiber diet could be a promising strategy to support the recovery of microbial communities when the animals are submitted to antibiotic stress.

In conclusion, the data presented in this study indicate that the broad-spectrum antibiotic TMS given orally for 5 days to horses heavily impacted the fecal bacteria diversity, structure, function, and metabolites. This is the first study to use a global and comprehensive approach for giving an overall view of dysbiosis in the equine hindgut and its impacts on the host’s health using non-invasive biomarkers. The microbiota appeared to reorganize rapidly for ensuring fiber fermentation and recovered progressively to a basal state 2 to 9 days after the end of TMS administration. Likely associated with the variations in bacterial communities, changes occurred in the immune mucosal and peripheral hindgut homeostasis during TMS administration. Supplementation with a combination of *L. salivarium*, *L. acidophilus*, and *B. lactis* might support the hindgut mucosal integrity and contribute to equine health. Although further studies are needed, new probiotic strategies are promising for ensuring fiber degradation and VFA absorption during antibiotic-induced dysbiosis and in avoiding adverse digestive effects.

## Data Availability Statement

The datasets presented in this study can be found in online repositories. The names of the repository/repositories and accession number(s) can be found in the article/Supplementary Material.

## Ethics Statement

The animal study was reviewed and approved by Committee on the Ethics of Animal Experiments of Grand Campus Dijon.

## Author Contributions

All authors designed the research study and conducted the research collectively. AC and PG collected the data. AC, SJ, and PG performed the statistical analyses. AC, PG, and VJ wrote the manuscript. SJ contributed to critical revisions of the manuscript. All authors approved the final version of the manuscript.

## Conflict of Interest

The authors declare that the research was conducted in the absence of any commercial or financial relationships that could be construed as a potential conflict of interest.
