# Supplementary material for: Multidimensional Approach for Investigating the Effects of an Antibiotic–Probiotic Combination on the Equine Hindgut Ecosystem and Microbial Fibrolysis
Source: Front Microbiol. 2021 Mar 25;12:646294. doi: 10.3389/fmicb.2021.646294 (PMC8027512; doi:10.3389/fmicb.2021.646294)
Supplement: Supplementary file 1 [file Data_Sheet_1.pdf]

**Supplementary Table 1.** Chemical and nutritional composition of the diet.

| Diet | |
|---|---|
| Digestible energy (kcal/100 kg BW) | 4800 |
| Dry Matter (DM) (g/100 g) | 91.92 |
| Hay proportion (% DM) | 14.17 |
| Concentrates proportion (% DM) | 85.83 |
| Hay ingestion level (% BW DM / day) | 1.88 |
| Concentrates ingestion level (% BW DM / day) | 0.30 |
| Starch ingestion level (% BW DM / day) | 0.10 |
| Starch (% DM) | 4.81 |
| Crude Protein (CP, % DM) | 9.71 |
| Neutral Detergent Fiber (NDF, % DM) | 56.76 |
| Acid Detergent Fiber (ADF, % DM) | 35.75 |
| Acid Detergent Lignin (ADL, % DM) | 4.38 |
| NDF – ADF / Hemicellulose (% DM) | 21.01 |
| ADF – ADL / Cellulose (% DM) | 31.38 |

**Supplementary Table 2.** Bacterial family relative abundance variations in the fecal microbiota of horses subject to an oral challenge with TMS from day (D)0 to D4. Only families with mean relative abundances greater than 0.1% of the total relative abundance in all the fecal samples and showing a significant day effect are presented.

| **Bacterial family name (Taxonomic ranks: Phylum > Class > Order)** | **D0*** | **D2** | **D7** | **D14** | **D21** | **D28** | **Mean ± S.D.** | **p-value** |
|---|---|---|---|---|---|---|---|---|
| **Anaeroplasmataceae** (Tenericutes>Mollicutes>Anaeroplasmatales) | 0.07$^{b}$ | 0.24$^{a}$ | 0.08$^{b}$ | 0.08$^{b}$ | 0.08$^{b}$ | 0.07$^{b}$ | 0.10 ± 0.24 | 0.0436 |
| **Bacteroidales UCG-001** (Bacteroidetes>Bacteroidia>Bacteroidales) | 1.34$^{a}$ | 0.85$^{b}$ | 1.09$^{ab}$ | 1.39$^{a}$ | 1.25$^{a}$ | 1.10$^{ab}$ | 1.17 ± 0.78 | 0.0377 |
| **Christensenellaceae** (Firmicutes>Clostridia>Clostridiales) | 2.58$^{b}$ | 3.50$^{a}$ | 2.52$^{b}$ | 2.41$^{b}$ | 2.62$^{b}$ | 2.41$^{b}$ | 2.67 ± 0.99 | 0.0001 |
| **Clostridiales vadinBB60 group** (Firmicutes>Clostridia>Clostridiales) | 0.22$^{b}$ | 0.35$^{a}$ | 0.26$^{ab}$ | 0.19$^{b}$ | 0.23$^{b}$ | 0.20$^{b}$ | 0.24 ± 0.21 | 0.0476 |
| **Defluviitaleaceae** (Firmicutes>Clostridia>Clostridiales) | 0.38$^{a}$ | 0.25$^{bc}$ | 0.19$^{c}$ | 0.34$^{ab}$ | 0.32$^{ab}$ | 0.33$^{ab}$ | 0.30 ± 0.31 | 0.0162 |
| **Eggerthellaceae** (Actinobacteria>Coriobacteriia>Coriobacteriales) | 0.18$^{ab}$ | 0.13$^{c}$ | 0.14$^{bc}$ | 0.16$^{abc}$ | 0.18$^{a}$ | 0.16$^{abc}$ | 0.16 ± 0.07 | 0.0363 |
| **Family XIII** (Firmicutes>Clostridia>Clostridiales) | 1.49$^{ab}$ | 1.59$^{a}$ | 1.27$^{bc}$ | 1.34$^{abc}$ | 1.51$^{ab}$ | 1.15$^{c}$ | 1.39 ± 0.52 | 0.0047 |
| **Fibrobacteraceae** (Fibrobacteres>Fibrobacteria>Fibrobacterales) | 2.83$^{ab}$ | 1.78$^{c}$ | 2.73$^{ab}$ | 2.39$^{abc}$ | 2.10$^{bc}$ | 2.92$^{a}$ | 2.46 ± 1.72 | 0.0242 |
| **gir-aah93h0** (Bacteroidetes>Bacteroidia>Bacteroidales) | 0.14$^{bc}$ | 0.38$^{a}$ | 0.31$^{ab}$ | 0.22$^{abc}$ | 0.09$^{c}$ | 0.13$^{bc}$ | 0.21 ± 0.40 | 0.0188 |
| **Lachnospiraceae** (Firmicutes>Clostridia>Clostridiales) | 24.65$^{a}$ | 22.36$^{b}$ | 22.61$^{b}$ | 24.97$^{a}$ | 24.94$^{a}$ | 25.12$^{a}$ | 24.11 ± 4.31 | 0.0131 |
| **Muribaculaceae** (Bacteroidetes>Bacteroidia>Bacteroidales) | 0.42$^{abc}$ | 0.44$^{ab}$ | 0.34$^{bc}$ | 0.31$^{c}$ | 0.35$^{bc}$ | 0.46$^{a}$ | 0.39 ± 0.27 | 0.0296 |
| **Paludibacteraceae** (Bacteroidetes>Bacteroidia>Bacteroidales) | 0.61$^{b}$ | 1.37$^{a}$ | 0.85$^{b}$ | 0.51$^{b}$ | 0.62$^{b}$ | 0.48$^{b}$ | 0.74 ± 0.88 | 0.0008 |
| **Prevotellaceae** (Bacteroidetes>Bacteroidia>Bacteroidales) | 11.14$^{c}$ | 14.78$^{a}$ | 12.55$^{b}$ | 12.00$^{bc}$ | 11.08$^{c}$ | 12.12$^{bc}$ | 12.28 ± 2.82 | <0.0001 |
| **Rikenellaceae** (Bacteroidetes>Bacteroidia>Bacteroidales) | 7.32$^{ab}$ | 5.68$^{c}$ | 7.10$^{ab}$ | 7.17$^{ab}$ | 8.01$^{a}$ | 6.59$^{bc}$ | 6.98 ± 2.13 | 0.0003 |
| **Ruminococcaceae** (Firmicutes>Clostridia>Clostridiales) | 20.80$^{ab}$ | 22.21$^{a}$ | 22.09$^{a}$ | 20.11$^{b}$ | 20.79$^{ab}$ | 19.26$^{b}$ | 20.88 ± 4.05 | 0.0157 |

* D0: Basal value before the first TMS administration.

**Supplementary Table 3**. Relative abundance variations in bacterial genera in the fecal microbiota of horses subject to TMS oral challenge from D0 to D4. Only genera with mean relative abundances greater than 0.1% of the total relative abundance in all the fecal samples and showing a significant day effect are presented.

| **Bacterial genus name**<br>**(Taxonomic ranks: Phylum > Class > Order > Family)** | **D0*** | **D2** | **D7** | **D14** | **D21** | **D28** | **Mean ± S.D.** | **p-value** |
|---|---|---|---|---|---|---|---|---|
| ***[Eubacterium] coprostanoligenes group***<br>(Firmicutes>Clostridia>Clostridiales>Ruminococcaceae) | 2.76$^{abc}$ | 3.05$^{ab}$ | 3.11$^{a}$ | 2.59$^{bc}$ | 2.71$^{abc}$ | 2.36$^{c}$ | 2.76 ± 1.07 | 0.0228 |
| ***[Eubacterium] ruminantium group***<br>(Firmicutes>Clostridia>Clostridiales>Lachnospiraceae) | 0.41$^{bc}$ | 0.23$^{d}$ | 0.34$^{cd}$ | 0.47$^{bc}$ | 0.53$^{ab}$ | 0.63$^{a}$ | 0.44 ± 0.32 | <0.0001 |
| ***Alloprevotella***<br>(Bacteroidetes>Bacteroidia>Bacteroidales>Prevotellaceae) | 0.51$^{c}$ | 1.18$^{a}$ | 0.93$^{ab}$ | 0.59$^{c}$ | 0.65$^{bc}$ | 0.52$^{c}$ | 0.73 ± 0.63 | <0.0001 |
| ***Anaeroplasma***<br>(Tenericutes>Mollicutes>Anaeroplasmatales>Anaeroplasmataceae) | 0.07$^{b}$ | 0.24$^{a}$ | 0.08$^{b}$ | 0.08$^{b}$ | 0.08$^{b}$ | 0.07$^{b}$ | 0.10 ± 0.24 | 0.0434 |
| ***Anaerovorax***<br>(Firmicutes>Clostridia>Clostridiales>Family XIII) | 1.02$^{a}$ | 0.90$^{ab}$ | 0.82$^{b}$ | 0.87$^{ab}$ | 1.00$^{a}$ | 0.77$^{b}$ | 0.90 ± 0.37 | 0.0287 |
| ***Christensenellaceae R 7 group***<br>(Firmicutes>Clostridia>Clostridiales>Christensenellaceae) | 2.54$^{b}$ | 3.45$^{a}$ | 2.48$^{b}$ | 2.36$^{b}$ | 2.58$^{b}$ | 2.37$^{b}$ | 2.63 ± 0.99 | 0.0001 |
| ***Defluviitaleaceae UCG-011***<br>(Firmicutes>Clostridia>Clostridiales>Defluviitaleaceae) | 0.38$^{a}$ | 0.25$^{bc}$ | 0.19$^{c}$ | 0.34$^{ab}$ | 0.32$^{ab}$ | 0.33$^{ab}$ | 0.30 ± 0.31 | 0.0161 |
| ***Desulfovibrio***<br>(Proteobacteria>Deltaproteobacteria>Desulfovibrionales>Desulfovibrionaceae) | 0.11$^{c}$ | 0.19$^{a}$ | 0.14$^{b}$ | 0.12$^{bc}$ | 0.12$^{bc}$ | 0.12$^{bc}$ | 0.13 ± 0.07 | <0.0001 |
| ***Family XIII AD3011 group***<br>(Firmicutes>Clostridia>Clostridiales>Family XIII) | 0.25$^{bc}$ | 0.38$^{a}$ | 0.26$^{bc}$ | 0.26$^{bc}$ | 0.28$^{b}$ | 0.19$^{c}$ | 0.27 ± 0.14 | <0.0001 |
| ***Fibrobacter***<br>(Fibrobacteres>Fibrobacteria>Fibrobacterales>Fibrobacteraceae) | 2.83$^{ab}$ | 1.78$^{c}$ | 2.73$^{ab}$ | 2.39$^{abc}$ | 2.10$^{bc}$ | 2.92$^{a}$ | 2.46 ± 1.72 | 0.0242 |
| ***Lachnoclostridium***<br>(Firmicutes>Clostridia>Clostridiales>Lachnospiraceae) | 0.63$^{b}$ | 1.00$^{a}$ | 0.69$^{b}$ | 0.64$^{b}$ | 0.62$^{b}$ | 0.71$^{b}$ | 0.72 ± 0.33 | <0.0001 |
| ***Lachnospiraceae AC2044 group***<br>(Firmicutes>Clostridia>Clostridiales>Lachnospiraceae) | 4.90$^{a}$ | 4.09$^{b}$ | 3.95$^{b}$ | 5.01$^{a}$ | 4.93$^{a}$ | 4.56$^{ab}$ | 4.57 ± 1.83 | 0.0350 |
| ***Mailhella***<br>(Proteobacteria>Deltaproteobacteria>Desulfovibrionales>Desulfovibrionaceae) | 0.14$^{a}$ | 0.09$^{bc}$ | 0.12$^{ab}$ | 0.14$^{a}$ | 0.11$^{abc}$ | 0.08$^{c}$ | 0.11 ± 0.08 | 0.0090 |
| ***Prevotella***<br>(Bacteroidetes>Bacteroidia>Bacteroidales>Prevotellaceae) | 3.68$^{b}$ | 4.33$^{a}$ | 4.05$^{ab}$ | 3.45$^{b}$ | 3.47$^{b}$ | 4.09$^{ab}$ | 3.85 ± 1.54 | 0.0384 |
| ***Prevotellaceae UCG-001***<br>(Bacteroidetes>Bacteroidia>Bacteroidales>Prevotellaceae) | 2.83$^{b}$ | 4.35$^{a}$ | 2.98$^{b}$ | 3.11$^{b}$ | 2.92$^{b}$ | 3.18$^{b}$ | 3.23 ± 1.76 | 0.0008 |
| ***Prevotellaceae UCG-004***<br>(Bacteroidetes>Bacteroidia>Bacteroidales>Prevotellaceae) | 1.21$^{c}$ | 1.65$^{a}$ | 1.45$^{ab}$ | 1.29$^{bc}$ | 1.18$^{c}$ | 1.20$^{c}$ | 1.33 ± 0.49 | 0.0008 |
| ***Rikenellaceae RC9 gut group***<br>(Bacteroidetes>Bacteroidia>Bacteroidales>Rikenellaceae) | 5.73$^{ab}$ | 4.23$^{c}$ | 5.39$^{ab}$ | 5.53$^{ab}$ | 6.20$^{a}$ | 4.98$^{bc}$ | 5.34 ± 1.98 | 0.0016 |
| ***Ruminococcaceae UCG-005***<br>(Firmicutes>Clostridia>Clostridiales>Ruminococcaceae) | 1.84$^{b}$ | 2.40$^{a}$ | 1.92$^{b}$ | 2.07$^{b}$ | 2.02$^{b}$ | 1.85$^{b}$ | 2.02 ± 0.57 | 0.0002 |
| ***Ruminococcaceae UCG-010***<br>(Firmicutes>Clostridia>Clostridiales>Ruminococcaceae) | 2.98$^{bc}$ | 4.12$^{a}$ | 3.61$^{ab}$ | 2.58$^{c}$ | 2.90$^{c}$ | 2.42$^{c}$ | 3.10 ± 1.43 | <0.0001 |
| ***Ruminococcaceae UCG-011***<br>(Firmicutes>Clostridia>Clostridiales>Ruminococcaceae) | 0.09$^{bc}$ | 0.09$^{bc}$ | 0.12$^{a}$ | 0.08$^{c}$ | 0.11$^{ab}$ | 0.08$^{c}$ | 0.10 ± 0.07 | 0.0377 |
| ***Ruminococcaceae UCG-014***<br>(Firmicutes>Clostridia>Clostridiales>Ruminococcaceae) | 0.63$^{a}$ | 0.37$^{b}$ | 0.52$^{a}$ | 0.58$^{a}$ | 0.64$^{a}$ | 0.56$^{a}$ | 0.55 ± 0.31 | 0.0056 |
| ***Saccharofermentans***<br>(Firmicutes>Clostridia>Clostridiales>Ruminococcaceae) | 1.19$^{a}$ | 0.78$^{b}$ | 0.98$^{ab}$ | 1.09$^{a}$ | 1.20$^{a}$ | 1.24$^{a}$ | 1.08 ± 0.55 | 0.0070 |

* D0: Basal value before the first TMS administration.

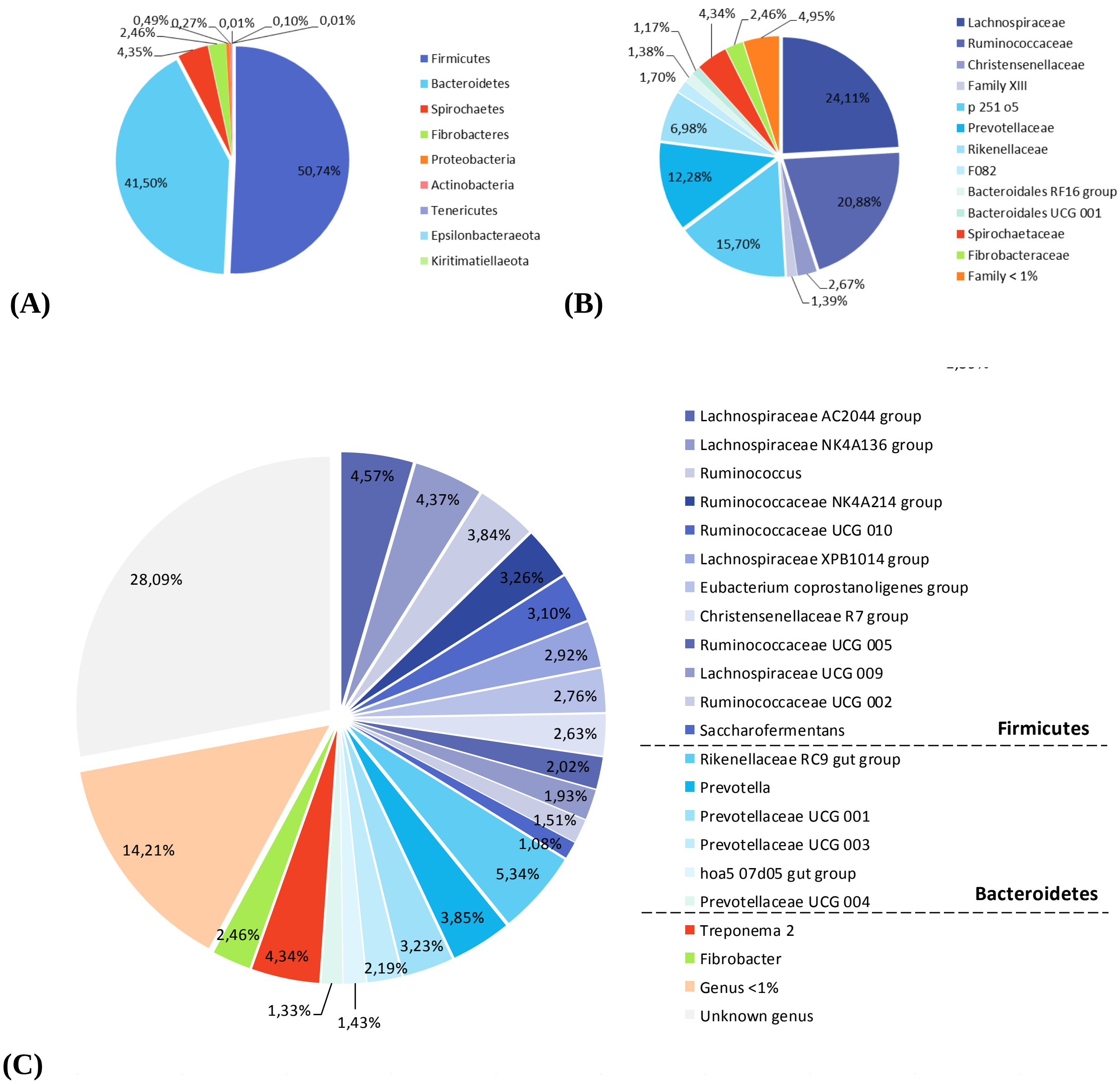


**Supplementary Figure 1**. Depiction of the mean relative abundances observed at phylum (A), family (B) and genus (C) levels in the fecal microbiota of horses, for all supplementation modalities during the whole experimental trial (abundances are shown as percentage of the total number of sequences).

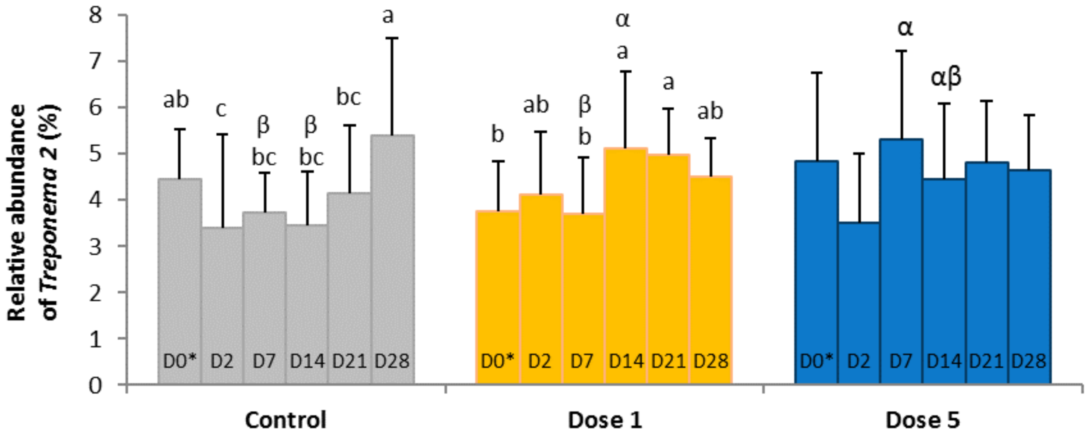


**(A)**

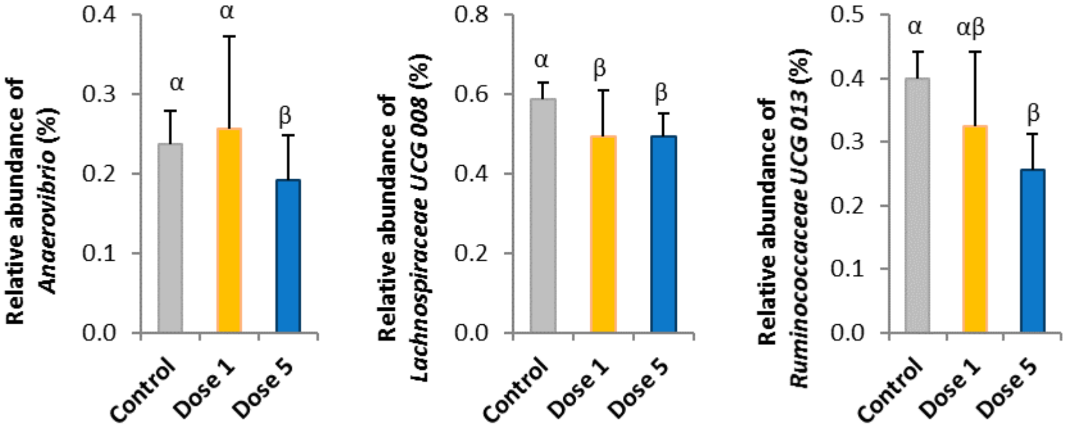


**(B)**

**Supplementary Figure 2**. Significant (A) day × supplementation interaction on Treponema 2 and (B) supplementation effects on *Anaerovibrio*, *Lachnospiraceae UGC 008* and *Ruminococcaeae UGC 013* in fecal samples of horses subjected to TMS oral challenge from D0 to D4. Different Latin letter superscripts show significant variations between days for each supplementation modality. Different Greek letter superscripts show significant variations between supplementations for each day. * D0: Basal value before the first TMS administration.

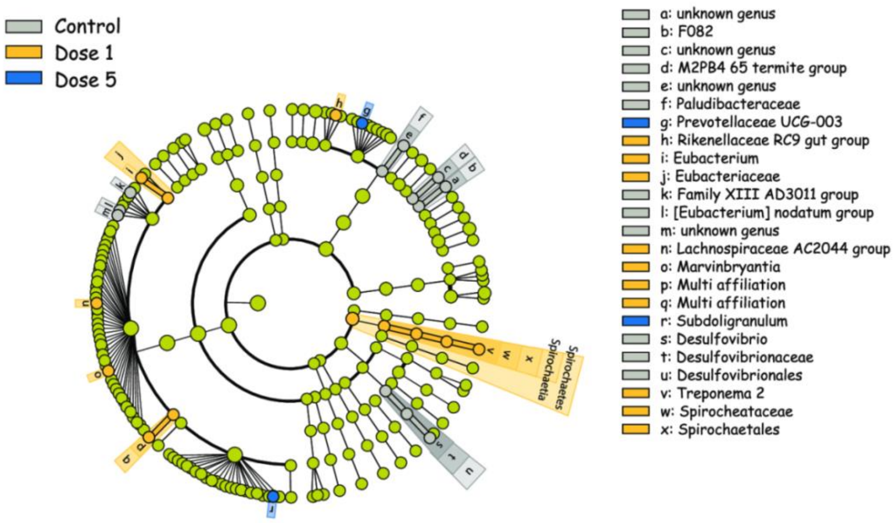


**Supplementary Figure 3.** LEfSe cladogram representing fecal equine bacterial microbiota categorized by the supplementation. The LEfSe cladogram comprises five concentric circles representing five taxonomic categories (phylum, class, order, family and genus) from the center to the periphery. Gray indicates enrichment in samples from control horses; orange and blue indicate taxa enriched in the samples from horses supplemented with Dose 1 and Dose 5, respectively.
